# Myeloid Sarcomas: A Clinicopathologic Study of 20 Cases

**DOI:** 10.4274/tjh.2013.0087

**Published:** 2015-02-15

**Authors:** Gülşah Kaygusuz, Duygu Kankaya, Cemil Ekinci, Pervin Topçuoğlu, Işınsu Kuzu

**Affiliations:** 1 Ankara University Faculty of Medicine, Department of Pathology, Ankara, Turkey; 2 Ankara University Faculty of Medicine, Department of Hematology, Ankara, Turkey

**Keywords:** Myeloid sarcoma, Granulocytic sarcoma, Monoblastic sarcoma

## Abstract

**Objective:**

Myeloid sarcoma is a tumoral mass of mature or immature myeloid blasts in extramedullary anatomic locations. It can be seen de novo or in association with acute myeloid leukemia, myeloproliferative neoplasias, or myelodysplastic syndrome. Isolated myeloid sarcoma can be seen as a relapse in cases with allogenic bone marrow transplantation. Although it may involve any tissue in the body, the most common locations are skin, soft tissues, lymph nodes, and the gastrointestinal tract. Immunohistochemically, most cases show myelomonocytic or pure monoblastic differentiation. We reviewed the clinicopathological features of 20 cases of myeloid sarcoma diagnosed in our institute in view of the literature.

**Materials and Methods:**

The cases diagnosed between 2005 and 2012 at the Ankara University Faculty of Medicine, Department of Pathology, were selected. Clinicopathological findings including the age and sex of the patients; symptoms; anatomic location; accompanying hematological disease; and the morphological, immunohistochemical, and cytogenetic features of the cases were noted.

**Results:**

Sixteen of the patients were male and 4 were female. The median age at diagnosis was 47 years. The most commonly involved locations were the lymph nodes and skin. Immunohistochemically, eleven cases were of the myelomonocytic and 7 cases were of the myeloid phenotype, whereas 2 cases showed pure monoblastic differentiation. The median follow-up period for the 18 cases with known clinical data was 33 weeks. Five patients died of the disease in an average of 36 weeks.

**Conclusion:**

Myeloid sarcoma is a rare presentation of leukemias, myeloproliferative neoplasias, or myelodysplastic syndrome, composed of immature myelomonocytic cells in extramedullary tissues. It may present with variable morphological and phenotypic features, always creating a challenge in pathological diagnosis.

## Abstract (tr)

**Amaç:**

Miyeloid sarkom, ekstramedüller anatomik bölgelerde matür veya immatür miyeloid blastların oluşturduğu tümöral kitledir. De novo veya akut miyeloid lösemi, miyeloproliferatif neoplaziler ya da miyelodisplastik sendrom ile birlikte görülebilir. Tek başına miyeloid sarkoma, allojenik kemik iliği transplantasyonu yapılan olgularda nüks şeklinde görülebilir. Vücutta herhangi bir dokuda gelişebilmekle birlikte en sık tutulan bölgeler deri, yumuşak dokular, lenf nodülü ve gastrointestinal sistemdir. İmmünohistokimyasal olarak olguların birçoğu miyelomonositik ya da saf monoblastik farklılaşma gösterir. Bu makalede kurumumuzda tanı alan 20 miyeloid sarkoma olgusunun klinikopatolojik özellikleri kaynaklar ışığında gözden geçirilmiştir.

**Gereç ve Yöntemler:**

2005-2012 yılları arasında Ankara Üniversitesi Tıp Fakültesi Patoloji departmanında tanı alan olgular seçilmiştir. Hastaların yaş, cinsiyet, semptomlar, anatomik yerleşim, eşlik eden hematolojik hastalık bilgileri ve olguların morfolojik, immünohistokimyasal ve sitogenetik özellikleri kayıt edilmiştir.

**Bulgular:**

On altı hasta erkek, 4 hasta kadındır. Tanı anındaki ortalama yaş 47’dir. En sık tutulan bölgeler lenf nodları ve deridir. İmmünohistokimyasal olarak 11 olguda miyelomonositik ve 7 olguda miyeloid fenotip, 2 olguda pür monoblastik farklılaşma saptanmıştır. Klinik verileri bilinen 18 olgu için ortalama takip süresi 33 hafta bulunmuştur. Hastalardan 5’i hastalığı nedeniyle kaybedilmiştir.

**Sonuç:**

Miyeloid sarkom, ekstramedüller dokularda immatür miyelomonositik hücrelerin oluşturduğu; lösemiler, miyeloproliferatif neoplaziler veya miyelodisplastik sendromların nadir bir prezentasyonudur. Daima patolojik tanı güçlüğü yaratacak şekilde değişken morfolojik ve fenotipik bulgularla ortaya çıkabilir.

## INTRODUCTION

Myeloid sarcoma (MS) is a tumoral mass composed of mature or immature myeloid blasts in extramedullary anatomic locations. There is a predilection for males with the median age of 56 years (1-89 years) [1]. MS can be seen de novo or in association with acute myeloid leukemia (AML), myeloproliferative neoplasias (MPNs), or myelodysplastic syndrome (MDS) [2,3,4,5,6]. It has been observed most commonly in pediatric AML cases (30%) and is less frequently seen among adult AML patients (2%-5%) [7]. MS can be the first manifestation of AML, or it can arise as a recurrence of treated AML. While it can be reported from any anatomic region, the most common locations are the skin, soft tissues, lymph nodes, and gastrointestinal tract [7]. Most cases show myelomonocytic or pure monoblastic differentiation. The differential diagnosis of MS includes a large group of entities according to the clinical history, age of the patient, or information on preexisting or concomitant myeloid neoplasia.

The clinicopathological features of 20 cases of MS are reviewed in this manuscript along with previously published reports.

## MATERIALS AND METHODS

The cases diagnosed as MS between 2005 and 2012 at the Ankara University Faculty of Medicine, Department of Pathology, were selected. Clinicopathological findings including the age and sex of the patients; symptoms; anatomic location; accompanying hematological disease; the morphological, immunohistochemical, and cytogenetic features of the cases; and the follow-up data are summarized in Table 1. Informed consent was obtained.

## RESULTS

Sixteen of the patients were male and 4 were female. The median age at diagnosis was 47 years (range: 22-82 years). In 7 cases, more than 1 anatomic region was involved. The most commonly involved locations were the lymph nodes (9 cases) and skin (3 cases), while the breast (2 cases), paratubal tissue (2 cases), and soft tissues of pectoral or lumbar (2 cases) regions were the next most commonly involved sites. The rest of the locations were as follows: thoracic vertebra (1 case), submandibular gland (1 case), minor salivary gland (1 case), pleura (1 case), anterior mediastinum (1 case), retroperitoneum (1 case), liver (1 case), small bowel (1 case), gingiva (1 case), and ovary (1 case) (Table 1).

The main symptoms of the patients were related to the site of the lesions. Regional pain (6 cases), weakness (4 cases), palpable mass (4 cases), lymphadenopathy (3 cases), weight loss (2 cases), fecal incontinence (1 case), ileus (1 case), skin rash (1 case), mouth sore (1 case), vomiting (1 case), and hypermenorrhea (1 case) were the recorded symptoms and complaints. Ten patients presented with preexisting or concurrent AML (1 case primary myelofibrosis-based) and 2 with chronic myeloid leukemia (CML). Three cases were presented as de novo disease (Table 1). In 5 of the cases there were no bone marrow or clinical data revealing the association of primary myeloid neoplasia. The disease developed following allogenic bone marrow transplantation in 3 of 13 cases. Tuberculosis, myxoid liposarcoma, and colonic adenocarcinoma were simultaneously diagnosed second pathologies in 3 MS cases.

**Figure 1:**
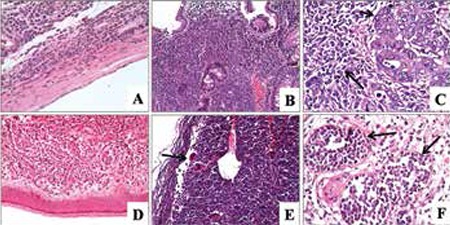
A, B, D) Diffuse blastic infiltration in the wall of a Morgagni cyst, in the small intestinal mucosa, and in the dermis (H&E 400x, 200x, 400x, respectively). C) Blastic cells (black arrow) intermingled with adenocarcinoma of the colon (H&E 400x). E) Case 16 showed sinusoidal infiltration of the blastic cells mixed with megakaryocytes (black arrow; H&E 400x). F) Angiolymphatic invasion was seen (black arrows; H&E 400x).

**Figure 2:**
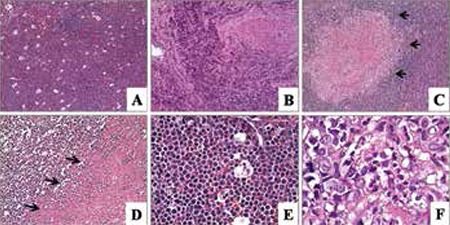
A, B) Starry sky appearance and crushing artifact were noted (H&E 100x). C) Granuloma with blastic cell infiltration (black arrows; H&E 100x). D, E) Necrosis (black arrows) and numerous mitoses were found (H&E 200x, 400x, respectively). F) Pleomorphic blastic cells (H&E 1000x).

In one case, blastic cells were noted in the wall of a Morgagni cyst (Figure 1A). A diffuse infiltration pattern was noted in other extranodal locations (Figures 1B and 1D). One case showed infiltration of adenocarcinoma of the colon intermingled with MS (case 15; Figure 1C). Morphologically, 6 of 9 cases were characterized by the complete effacement of the lymph node, while 1 case had paracortical infiltration (case 11) and 1 case had sinusoidal infiltration (case 16) (Figure 1E). Morphological signs of aggressive behavior of the tumors, such as angiolymphatic invasion (1 case; Figure 1F), starry sky appearance (Figure 2A; 1 case), crushing artifacts (2 cases; Figure 2B), necrosis (2 cases; Figure 2C, 2D), and marked nuclear pleomorphism (1 case; Figure 2E, 2F), were present on histopathological examination (Table 1). Non-necrotic granulomas were observed in a case showing sinusoidal infiltration of the lymph node (Figure 2C). In almost all of the tumors, the mitotic rate was high (Figure 2E).

**Figure 3:**
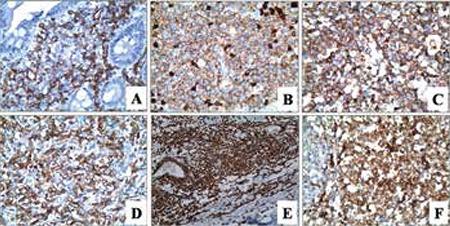
Immunohistochemical CD34 (A), CD45 (B), MPO (C), CD117 (D), Tdt (E), and CD68 (F) expression of the neoplastic cells (400x, 400x, 400x, 400x, 200x, 400x, respectively).

Immunohistochemically, variable expressions of myeloid markers myeloperoxidase (MPO), CD34, CD68/PG-M1, CD117, lysozyme, CD45, terminal deoxynucleotidyl transferase (TDT), CD4, CD15, CD56, CD31, CD10, and Bcl2 were observed (Figures 3A-3F). Eleven cases were of the myelomonocytic and 7 cases were of the myeloid phenotype, whereas 2 cases showed pure monoblastic differentiation (Table 1).

Cytogenetic analyses were routinely performed from bone marrow aspirates. Cytogenetic data were present for 10 cases in our series. The 4 cases with positive cytogenetic abnormalities all had concurrent bone marrow involvement at diagnosis of MS (Table 1): 1 case with t(8;21) (q22;q22) (case 18); 1 case with FLT3-ITD heterozygous mutation and del(22q11) (case 12); 1 case with t(9;22) (q34;q11), inv(17) (q10), and trisomy 8 (case 10); and 2 cases with t(9;22) (q34;q11). Although 4 of them had concurrent AML with MS and 2 cases were de novo, there were no cytogenetic abnormalities detected in 6 cases. Cytogenetic data were not available in 10 cases.

The median follow-up period for the 18 cases with known clinical data was 33 weeks (range: 1-144 weeks). Five patients died of the disease in an average of 36 weeks (range: 1-80 weeks) (Table 1).

## DISCUSSION

MS, also known as granulocytic/monoblastic sarcoma or extramedullary myeloid tumor, was first known as chloroma, with its macroscopically greenish color caused by production of myeloperoxidase [8]. It occurs most often in males at the median age of 56 years (range: 1-89 years) [1]. Male sex predominance was also reported with a median age of 47, ranging between 22 and 82 years. Besides its most common locations in the skin, soft tissue, lymph nodes, and gastrointestinal tract, MS may involve any organ or system in the body [7]. The symptoms are substantially related with the anatomic location that is involved. The lymph nodes were the most frequent localizations involved in our series.

MS may develop as de novo disease, or it may occur in combination with myeloid neoplasias (AML, MPNs, or MDS) in 1/3 of the reported cases [2,3,4,5,6]. MS can be the first manifestation of AML, or it can arise as a relapse of treated AML. It may occur as a first sign of blastic crisis in CML [9]. Pileri et al. reported that 4 of 92 MS cases had preexisting or concurrent primary myelofibrosis (PMF) in their series [3]. Neiman et al. reported MS development in 5% of PMF cases in their series [10]. In this presented series, we have seen 10 cases of AML, 1 of PMF and 2 that were CML-based. Three of our patients presented with de novo disease. It has been suggested that the bone marrow involvement can be determined in approximately 10 months in all de novo MS cases [9]. Our 3 de novo patients were lost 1 to 4 weeks following their diagnoses. During their short follow-up period, bone marrow involvement could not be demonstrated. MS developed within an average of 25 months (range: 1-84 months) in cases of preexisting AML.

Isolated MS can be seen as the recurrent form of the disease in cases with allogenic bone marrow transplantation. Clark et al. reported the frequency as 8%-20% in their series [11]. Three of the MS patients in our series had recurrence of the disease during their follow-up period after allogenic bone marrow transplantation. 

Tuberculosis, myxoid liposarcoma, and colonic adenocarcinoma were coincidentally present in 3 MS cases in the current series. Rare cases with association of MS and colon adenocarcinoma were previously reported by Rocca et al., proposing the association with p53 deletion in intestinal stem cells [12].

Myeloid sarcomas are immature cell proliferations that are morphologically composed of myeloblasts, monoblasts/promonocytes, and rarely promyelocytes. A variable number of mature myeloid cells may be present. Necrosis, numerous mitoses, and tingible-body macrophages can be present [3]. The neoplastic cells may infiltrate lymph nodes in an intrasinusoidal or diffuse pattern. In extranodal localizations, the infiltration pattern is generally diffuse, or the tumor cells constitute cohesive groups in a sclerotic stroma, resembling metastatic carcinoma. In our series, 6 of 9 cases were characterized by the complete effacement of the normal lymph node structure, while 1 case had paracortical and 1 case had sinusoidal infiltration. All of these patterns may create difficulty at diagnosis in de novo cases. Non-necrotic granulomas were present in the case with nodal sinusoidal infiltration, creating a diagnostic difficulty. The patients’ clinical history did not reveal previously defined tuberculosis or any other granulomatous disease. Granulomatous reaction is rarely seen in conjunction with MS. Baksh et al. reported a case of leukemia cutis with granulomatous reaction, suggesting that is caused by the host response to damaged elastin fibers and other tissue stromal elements produced by leukemic infiltration [13].

MSs were also called granulocytic sarcoma and monoblastic sarcoma in the past, according to the morphological features [1,14]. This morphological classification was abandoned with molecular and cytogenetic classifications. A majority of cases have myelomonocytic or pure monoblastic morphology and immunophenotype. MS with erythroid and megakaryoblastic differentiation or extramedullary acute promyelocytic leukemia rarely occurs [15,16,17,18]. Immunophenotypic characteristics of MSs are also parallel to the morphological features. Various expressions of CD68/KP1, MPO, CD117, CD99, CD68/PG-M1, and lysozyme, concordant with the myelomonocytic differentiation, can be seen. Other than these markers, CD34, TdT, CD56, CD30, glycophorin A, and CD4 are also useful for diagnosis [19]. CD43 and lysozyme are sensitive but not specific for the diagnosis. CD68 and MPO are commonly expressed, whereas CD34 is negative, especially in cases with monocytic differentiation [20,21]. Variable CD45 expression can be seen, and CD33 and CD117 can be used to demonstrate myeloid nature [22,23]. Parallel with the previous reports, most of our patients carried the myelomonocytic (11 cases) or myeloid (7 cases) phenotypes, whereas 2 showed pure monoblastic differentiation.

Cytogenetic abnormalities, particularly monosomy 7, trisomy 8, MLL rearrangement, and inv(16), can be seen in about 55% of MS cases. t(8;21) (q22;q22) occurs in the pediatric age group more so than in adults [1]. In our series, cytogenetic analyses revealed 1 case with t(8;21) (q22;q22); 1 case with FLT3-ITD heterozygous mutation and del(22q11); 1 case with t(9;22) (q34;q11), inv(17) (q10), and trisomy 8; and 2 cases with t(9;22) (q34;q11). There were no cytogenetic changes in 6 cases. Conventional cytogenetics cannot be used as a routine procedure for MS, especially for de novo cases before the diagnosis. Performing cytogenetic examination for MS can be difficult if there is no bone marrow or peripheral blood involvement. It is difficult to speculate about the cytogenetic results in our series as we obtained very limited data. In 4 cases, although there was concurrent AML, conventional cytogenetics did not give any abnormalities. These cases could represent AML without any cytogenetic abnormalities.

The differential diagnosis of MS can be especially difficult in small biopsies. In this category, MS should be differentiated from lymphomas, particularly diffuse large B-cell lymphoma, lymphoblastic lymphoma, blastic variants of mantle cell lymphoma, and Burkitt’s lymphoma. The morphological diagnosis of MS can be easier if an extramedullary hematopoiesis picture is present with erythroid precursors, megakaryocytes, and eosinophilic myelocytes mixed with neoplastic cells. Immunohistochemically, the expression of B-cell markers such as CD20 and CD79a is rarely seen in MS, but it should be noted that cases with t(8;21) (q22;q22) may express PAX5, CD19, and CD79a [14,19]. Since MS may express T-cell markers (such as CD43, CD45, CD4, and CD7), immunohistochemical expressions of MPO, lysozyme, and CD68 should be analyzed for their distinction from T-cell lymphomas [24].

The cutaneous neoplastic infiltrates that are positive for CD56 and CD4 should be differentiated from blastic plasmacytoid dendritic cell neoplasms. The presence of MPO and lysozyme expression as well as negative T-cell leukemia 1 staining favors the diagnosis of MS [25].

Histiocytic sarcomas are rare tumors, which may be seen in extranodal sites and have common clinicopathological features with AML. Negative staining with CD33 and CD13 is helpful for their diagnosis. Malignant melanomas and poorly differentiated carcinomas are the other tumors that need to be differentiated from MS. Immunohistochemistry with S100, Melan-A, HMB-45, and AE1/AE3 can be helpful in providing the correct diagnosis.

Since non-monocytic MSs are mostly CD99-positive, other blue round cell tumors of the pediatric age such as Ewing sarcoma/PNET or medulloblastoma may mimic MS. The myeloid and monocytic markers may help in diagnosis, but flow cytometric analysis of the specimen should be performed [20].

Studies concerning the prognosis and therapy response of MSs are limited. The therapy options are local radiotherapy, systemic chemotherapy, immunotherapy, and donor lymphocyte infusion for the allogenic transplanted cases [11]. Patients are generally treated with induction chemotherapy and allogenic/autologous bone marrow transplantation. Success in therapy in the long term is very low (10%), and the mortality associated with therapy is high (>60%) [12]. In our series, the mean follow-up period was 33 weeks (range: 1-144 weeks) for 18 of the patients with known follow-up data. Five of those died from the disease within an average of 36 weeks (range: 1-80 weeks) following their diagnosis.

In conclusion, MS is a rare presentation of leukemias, myeloproliferative neoplasias, or myelodysplastic syndrome, composed of immature myelomonocytic cells in extramedullary tissues. It may present with variable morphological and phenotypic features, always creating a challenge in pathological diagnosis. In addition to its diagnostic difficulty, overcoming the disease is also crucial, as it results in poor survival. 

## Figures and Tables

**Table 1:**
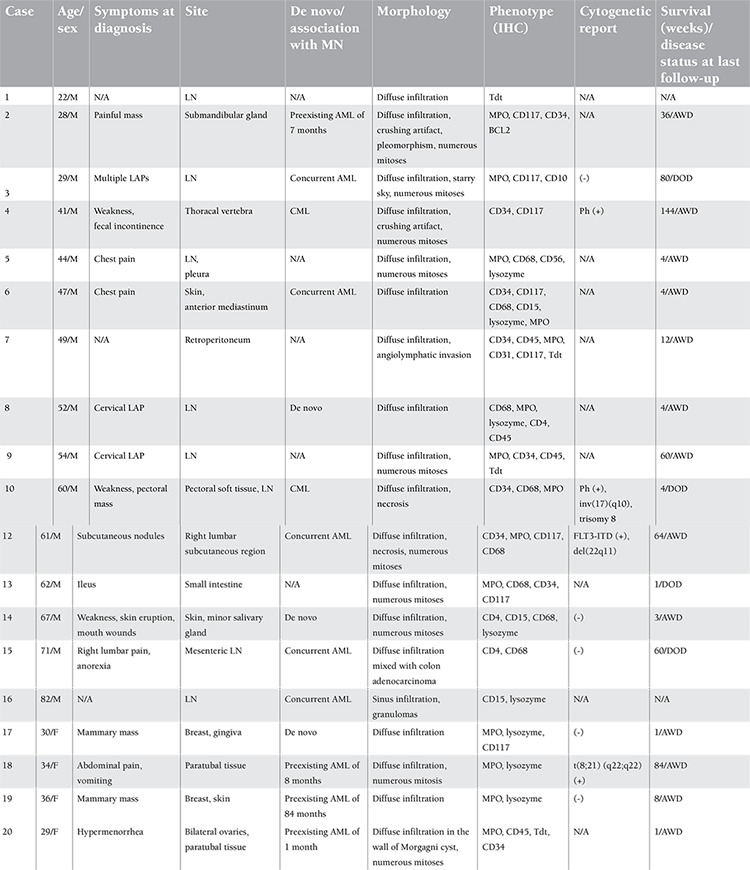
The clinicopathological features of the cases.
